# A Bivalent COVID-19 Vaccine Based on Alpha and Beta Variants Elicits Potent and Broad Immune Responses in Mice against SARS-CoV-2 Variants

**DOI:** 10.3390/vaccines10050702

**Published:** 2022-04-29

**Authors:** Rui Wang, Chunyun Sun, Juan Ma, Chulin Yu, Desheng Kong, Meng Chen, Xuejie Liu, Dandan Zhao, Shuman Gao, Shuyuan Kou, Lili Sun, Zeyong Ge, Jun Zhao, Kuokuo Li, Tao Zhang, Yanjing Zhang, Chunxia Luo, Xuefeng Li, Yang Wang, Liangzhi Xie

**Affiliations:** 1 Beijing Engineering Research Center of Protein and Antibody, Sinocelltech Ltd., Beijing 100176, China; rui_wang@sinocelltech.com (R.W.); chunyun_sun@sinocelltech.com (C.S.); juan_ma@sinocelltech.com (J.M.); chulin_yu@sinocelltech.com (C.Y.); desheng_kong@sinocelltech.com (D.K.); meng_chen@sinocelltech.com (M.C.); eminent_1124@126.com (X.L.); dandan_zhao@sinocelltech.com (D.Z.); shuman_gao@sinocelltech.com (S.G.); shuyuan_kou@sinocelltech.com (S.K.); lili_sun2@sinocelltech.com (L.S.); gzy1684075@163.com (Z.G.); bigvetzhaojun@126.com (J.Z.); likuok@sina.cn (K.L.); tao_zhang@sinocelltech.com (T.Z.); yanjing_zhang@sinocelltech.com (Y.Z.); chunxia_luo@sinocelltech.com (C.L.); xuefeng_li@sinocelltech.com (X.L.); yang_wang@sinocelltech.com (Y.W.); 2 Cell Culture Engineering Center, Chinese Academy of Medical Sciences & Peking Union Medical College, Beijing 100176, China

**Keywords:** bivalent, B.1.351, B.1.1.7, broad-spectrum neutralization, Th1-biased

## Abstract

With the emergence and rapid spread of new pandemic variants, especially variants of concern (VOCs), the development of next-generation vaccines with broad-spectrum neutralizing activities is of great importance. In this study, SCTV01C, a clinical stage bivalent vaccine based on trimeric spike extracellular domain (S-ECD) of SARS-CoV-2 variants Alpha (B.1.1.7) and Beta (B.1.351) with a squalene-based oil-in-water adjuvant was evaluated in comparison to its two corresponding (Alpha and Beta) monovalent vaccines in mouse immunogenicity studies. The two monovalent vaccines induced potent neutralizing antibody responses against the antigen-matched variants, but drastic reductions in neutralizing antibody titers against antigen-mismatched variants were observed. In comparison, the bivalent vaccine SCTV01C induced relatively higher and broad-spectrum cross-neutralizing activities against various SARS-CoV-2 variants, including the D614G variant, VOCs (B.1.1.7, B.1.351, P.1, B.1.617.2, B.1.1.529), variants of interest (VOIs) (C.37, B.1.621), variants under monitoring (VUMs) (B.1.526, B.1.617.1, B.1.429, C.36.3) and other variants (B.1.618, 20I/484Q). All three vaccines elicited potent Th1-biased T-cell immune responses. These results provide direct evidence that variant-based multivalent vaccines could play important roles in addressing the critical issue of reduced protective efficacy against the existing and emerging SARS-CoV-2 variants.

## 1. Introduction

Coronavirus disease 2019 (COVID-19) was first reported in December 2019 [1] and has become a global pandemic since then. As of 26 January 2022, a total of 360 million confirmed cases and a total of 5.6 million deaths have been confirmed worldwide [2]. Multiple vaccines based on the ancestral strain of SARS-CoV-2 have been approved, and vaccine coverage has reached over 70% in many regions and countries; however, herd-immunity has not been reached due to waning immunity over time [3,4,5,6,7] and the emergence of new variants [8,9,10,11,12,13,14,15,16].

Decreases in protective efficacies against emerging variants were observed in clinical trials and real-world evidence studies of first-generation COVID-19 vaccines [17,18,19,20,21,22,23,24].

SARS-CoV-2 virus is composed of nuclear material (RNA genome) surrounded by coat proteins including the spike (S) glycoprotein, which is glycosylated and homotrimeric in nature [25]. The receptor-binding domain (RBD) is located in the S1 subunit of the spike protein, which binds to the ACE2 receptor and mediates virus entry [26]. The genomes of SARS-CoV-2 are prone to mutation, with a speed of two mutations per month [27]. Evolution of the SARS-CoV-2 genome, driven by natural selection or vaccine-resistant mutations, might result in alterations in antigenicity and infectivity [10,28]. Therefore, it is of urgency and significance to develop second-generation vaccines that can stimulate broad-spectrum neutralizing antibodies against existing and future emerging variants of SARS-CoV-2 virus. Monovalent vaccines containing antigens from a single variant strain may not be sufficient to induce cross-protection to other existing and future emerging variants. Multivalent vaccines, a traditional approach providing broad coverage against antigenically variable pathogens, might overcome this obstacle.

To evaluate the potential advantages of multivalent vaccines over monovalent vaccines in broad-spectrum neutralizing capacity, we designed a bivalent vaccine SCTV01C based on the trimeric spike protein extracellular domain (S-ECD) proteins of variants Alpha (B.1.1.7) and Beta (B.1.351), adjuvanted with SCT-VA02B, a squalene-based oil-in-water emulsion similar to adjuvant systems 03 (AS03) and MF59^®^. The immunogenicity of SCTV01C was evaluated in naïve mice in comparison to its two individual antigen components as monovalent vaccines. Comparative studies of SCTV01C or a D614G variant-based monovalent vaccine (mimicking first-generation vaccines targeting the ancestral strain of SARS-CoV-2) as booster shots were also carried out in this study.

Further clinical studies of SCTV01C are ongoing to evaluate its application in unvaccinated populations and as booster doses for previously vaccinated populations. Future development of multivalent vaccines based on the same platform approach can be rapidly deployed to address potential risks posed by evolving SARS-CoV-2 variants.

## 2. Materials and Methods

### 2.1. Vaccine Antigens

SARS-CoV-2 S-ECD was fused to a T4 bacteriophage fibritin motif (i.e., T4 Foldon) to stabilize the trimeric protein conformation [29] produced in CHO cells and purified as vaccine antigens. Briefly, the DNA fragments encoding S-ECD (amino acid 1 to 1208) of SARS-CoV-2 variant Alpha or Beta with the fusion of T4-foldon in the C-terminal were synthesized and constructed into pD2535nt-HDP expression vector. The plasmid was transfected into Chinese hamster ovary (CHO) cells, followed by glutamine synthetase (GS)-based selection. For protein purification, the collected supernatants were captured by cation exchange chromatograph. The eluting peaks were then polished by anion-exchange chromatography and mixed anion-exchange chromatography. Finally, the penetration peak of mixed anion-exchange chromatography was ultra-filtrated to citrate buffer. Preclinical characterization showed the antigens to be 12 × 20 nm nanoparticles with high purities and excellent thermal stability [30]. SCTV01C contains a 1:1 mixture of the trimeric protein antigens of Alpha (B.1.1.7) and Beta (B.1.351) variants.

### 2.2. Vaccine Immunization in Animals

All animals used in this study were raised and cared for in facilities accredited by the Association for the Assessment and Accreditation of Laboratory Animal Care (AAALAC). All experimental procedures were conducted according to Chinese animal use guidelines and were approved by the Institutional Animal Care and Use Committee (IACUC). Female BALB/c and C57BL/6J mice (6–8 weeks old) were purchased from Beijing Vital River Laboratory Animal Technology Co. (Beijing, China). Antigens adjuvanted with SCT-VA02B (2 mg/dose) were used for immunization. A monovalent vaccine based on trimeric S-ECD of the first dominant variant (D614G variant), which shared the same sequence in the receptor-binding domain (RBD) and N-terminal domain (NTD) with the ancestral strain, was used to mimic the ancestral strain-based vaccines in immunogenicity studies of SCTV01C as booster shots.

In the single-dose immunization study, BALB/c mice (n = 10/group) were immunized with a single dose of B.1.1.7 monovalent vaccine, B.1.351 monovalent vaccine, or SCTV01C on day 0 at five different dose levels (0.5, 1, 2, 4 and 8 μg). Pseudovirus neutralizing activities of immune sera with a dilution ratio of 1:80 were analyzed on day 28.

In the prime-boost immunization study, C57BL/6J mice (n = 8/group) were immunized with B.1.1.7 monovalent vaccine (1 μg/dose), B.1.351 monovalent vaccine (1 μg/dose), or SCTV01C (1 μg/dose for each antigen) on day 0, 14 and 28. Antigen-specific total IgG titers and neutralizing antibody titers in mouse serum were analyzed on day 21. Cellular immune responses were analyzed on day 42.

To evaluate the immunogenicity of SCTV01C as booster shots, C57BL/6J mice (n = 6/group) were immunized with 2 doses of D614G vaccine (0.5 μg/dose) on day 0 and 21. On day 133 and 147, mice received two booster shots of SCTV01C (Mix Regimen; 0.5 μg/dose/antigen) or the D614G vaccine (D614G Regimen; 0.5 μg/dose). Neutralizing antibody titers were analyzed on day 147 and 161.

### 2.3. Antigen-Specific Antibody ELISA Assay

Antigen-specific antibody in mouse serum was detected by ELISA. Briefly, 5 μg/mL of antigen was coated into ELISA plate overnight at 4 °C. After blocking with 2% BSA-TBST buffer, 100 μL of diluted serum samples was transferred to the plates and incubated for 1 h. Anti-mouse IgG F(ab)2-HRP secondary antibody (Jackson ImmunoResearch, West Grove, PA, USA) at a dilution ratio of 1:5000 was incubated at room temperature for 1 h. For Antigen-specific mouse IgG1 and IgG2a analyses, R-mIgG1-R020-HRP (Sino Biological Inc., Beijing, China) or anti-mouse mIgG2c/HRP (Thermo Fisher Scientific, Waltham, MA, USA) were used as secondary antibody.

Plates were washed 5 times, and color development reaction was carried out by adding TMB substrates, and stopped by 2M H_2_SO_4_. Optical absorbance at 450 nm (OD_450_ nm) was read in a microplate reader (BioTek). Antibody titer was defined as the highest serum dilution ratio that causes OD450 nm to be 2.1 times higher than the background value of unimmunized mouse serum samples.

### 2.4. SARS-CoV-2 Pseudovirus-Based Neutralization Assay

SARS-CoV-2 pseudovirus was manufactured by Sinocelltech (Beijing, China). Huh-7 cell was obtained from Chinese Culture Tissue Collection Center (CCTCC, China). Luciferase Assay System (E1501) and Passive Lysis 5× Buffer (E1941) were purchased from Promega (Madison, WI, USA). For the quantitative measurement of neutralization antibodies, the SARS-CoV-2 pseudovirus PsV-Luc-Spike carrying the firefly luciferase gene was used. Mutations in S-ECD of each variant pseudoviruses are shown in Table 1. Briefly, vaccine-immunized serum samples (heat-inactivated at 56 °C for 30 min) were serially diluted, incubated with 200 TCID_50_/well pseudovirus (1 h at 37 °C, in a 5% CO_2_ incubator), and co-cultured with 2 × 10^4^ Huh-7 cells for 20 h. Relative light unit (RLU) was measured to evaluate luciferase activity (CentroXS^3^ LB 960 Microplate Luminometer). The calculation formula for the inhibition rate of the pseudovirus entry is: Inhibition (%) = (Postive RLU-Sample RLU)/(Postive RLU-Negative RLU) × 100%. The neutralizing antibody titer (50% inhibitory dilution, NAT_50_) is defined as the serum dilution at which the RLUs were reduced by 50% when compared with the positive control wells. Positive neutralizing antibody (NAb) was determined as greater than 50% inhibition, and the dose required to achieve this effect in 50% of the animals (ED_50_) was calculated.

### 2.5. Detection of Th1 and Th2 Cytokines

Antigen-Specific T-cell responses were quantified with IFNγ and IL-4 enzyme-linked immunospot (ELISpot) kit. Mouse IFN-gamma ELISpot PLUS (ALP), strips (3321-4AST-2) and Mouse IL-4 ELISpot PLUS (ALP) (3311-4APW-2) were purchased from Mabtech (Nacka, Sweden). For in vitro stimulation of mouse splenocytes, 15-mer peptides with 11 overlapping amino acids covering the entire SARS-CoV-2 spike protein were used (SciLight Biotechnology, Beijing, China), since 90% of the CD4^+^ T cell epitopes and 97% of the CD8^+^ T cell epitopes are conserved in the Alpha and Beta variants when compared with the ancestral strain [31]. Briefly, the isolated mouse splenocytes were cultured into ELISpot plate at a density of 2 × 10^5^ cells/100 μL per well. Then, Spike S peptides at a concentration of 2 μg/mL, with a volume of 100 μL per well, were added (or not, for negative control wells without stimulation). Cells were incubated for about 20 h, and then IFNγ or IL-4-positive cells were measured. The spots were counted in the Enzyme Linked Spot Analyzer (ImmunoSpot^®^ S6, CTL, Cleveland, OH, USA). The negative control value was used to subtract the background from the sample value. Values below zero were presented as zero. Data analysis was carried out with GraphPad Prism.

Th1 (IFNγ, IL-2) and Th1 (IL-4, IL-5 and IL-13) cytokines in culture supernatants from S peptides-stimulated mouse splenocytes were further evaluated with LegendPlex™ mouse Th1/Th2 Panel CBA kit (741054) according to manufacturer’s instructions (Biolegend, San Diego, CA, USA) and analyzed by flow cytometry (FACS Celesta, BD).

### 2.6. Statistical Analysis

Statistical analysis in this study was performed with GraphPad Prism (version 8.2.1, GraphPad Software, San Diego, CA, USA). Data concerning antibody titers, which followed the skew normal distribution, were presented as geometric mean titer (GMT) ± standard deviation (SD) and were log transformed, resulting in a normal distribution of the data, and then analyzed by unpaired, two-tailed Student’s *t* test. Other data in this paper met the criteria of normal distribution, were presented as Mean  ±  SD and analyzed using unpaired, two-tailed Student’s *t* test. *p*  <  0.05 was considered statistically significant.

## 3. Results

### 3.1. Humoral Immune Responses Induced by the B.1.1.7 Monovalent, B.1.351 Monovalent and SCTV01C Bivalent Vaccines after a Single Immunization in Mice

**Figure 1:**
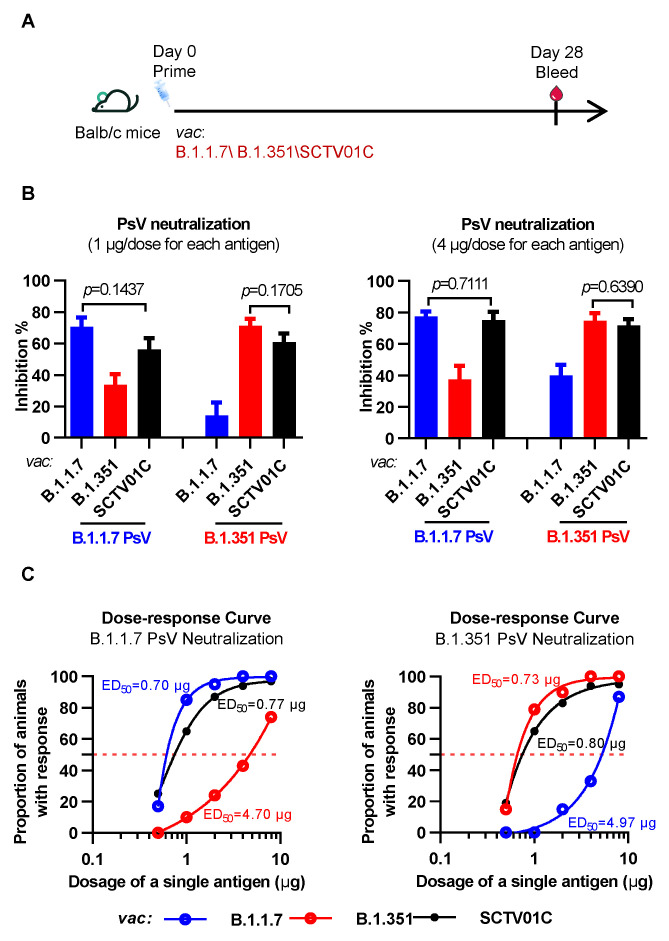
Humoral immune responses induced by the B.1.1.7 monovalent, B.1.351 monovalent and SCTV01C bivalent vaccines after a single-dose immunization. (**A**) Scheme of immunization and serum collection. BALB/c mice (n = 10/group) were immunized with a single dose of B.1.1.7 monovalent vaccine, B.1.351 monovalent vaccine, or SCTV01C on day 0 at five different dose levels (0.5, 1, 2, 4 and 8 μg for each antigen). Pseudovirus-neutralizing activities of immune sera with a dilution ratio of 1:80 were analyzed on day 28. (**B**) Inhibition rates of immune serum against the B.1.1.7 and B.1.351 PsV were calculated as described in the Materials and Methods section. (**C**) Positive neutralizing antibodies (NAb) were determined as greater than 50% inhibition, and the dose required to achieve this effect in 50% of the animals (ED_50_) was calculated. The results are representative of 3 independent experiments. Vac is an abbreviation for Vaccine. Bars show mean ± SD. Comparisons were performed by Student’s *t* test (unpaired, two-tailed).

The immunogenicity and dose response of the bivalent vaccine SCTV01C were first evaluated with a one-dose immunization scheme. BALB/c mice were injected with a single dose of trimeric B.1.351 S-ECD monovalent vaccine, trimeric B.1.1.7 S-ECD monovalent vaccine, or SCTV01C at five dose levels (0.5, 1, 2, 4 and 8 μg). Four weeks after immunization, serum samples were analyzed for neutralizing activities against the B.1.1.7 and B.1.351 variants using pseudovirus (PsV) neutralization assays (Figure 1A).

As shown in Figure 1B, the bivalent vaccine SCTV01C showed strong inhibition activities against both B.1.1.7 and B.1.351 variants. Compared with the B.1.1.7 monovalent vaccine, SCTV01C demonstrated a similar inhibition activity against the B.1.1.7 variant but a significantly higher inhibition activity against the B.1.351 variant. Similarly, SCTV01C showed significantly increased inhibition activity against the B.1.1.7 variant and equivalent activity against the B.1.351 variant when compared with the B.1.351 monovalent vaccine. Clearly, both monovalent vaccines showed significantly reduced cross-neutralizing titers to the non-vaccine variant when compared to the bivalent vaccine SCTV01C as expected.

Dose responses of SCTV01C and the two monovalent vaccines were analyzed. The dose required to achieve greater than 50% inhibition in the PsV neutralization assay in 50% of the mice was defined as the median effective dose (ED_50_) as a measure of vaccine potency (Figure 1C).

SCTV01C showed a similar dose–response curve and comparable ED_50_ value in the B.1.351 PsV assay as compared to the B.1.351 monovalent vaccine, and a similar dose–response curve and ED_50_ value in the B.1.1.7 PsV assay when compared to the B.1.1.7 monovalent vaccine. As expected, the B.1.1.7 monovalent vaccine showed a much weaker immune response against the non-vaccine variant B.1.351 (ED_50_ = 4.97 μg), and the B.1.351 monovalent vaccine showed a weak immune response against the non-vaccine variant B.1.1.7 (ED_50_ = 4.70 μg). A six-fold higher dose of either monovalent vaccine was needed to reach equivalent neutralizing potency against the non-vaccine variant when compared with SCTV01C.

### 3.2. Humoral Immune Responses Induced by the B.1.1.7 Monovalent, B.1.351 Monovalent and SCTV01C Bivalent Vaccines with a Prime-Boost Regimen in Mice

**Figure 2:**
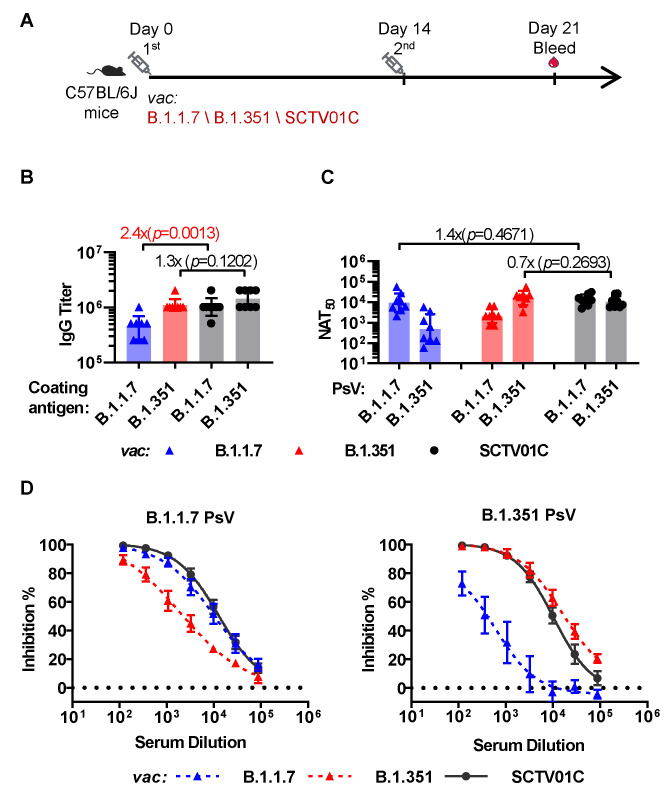
Humoral immune responses induced by the B.1.1.7 monovalent, B.1.351 monovalent and SCTV01C bivalent vaccines with a prime-boost regimen. (**A**) Scheme of immunization and serum collection. C57BL/6J mice (n = 8/group) were immunized with B.1.1.7 monovalent vaccine (1 μg/dose), B.1.351 monovalent vaccine (1 μg/dose), or SCTV01C (1 μg/dose for each antigen) on days 0 and 14. Humoral immune responses on day 21 were analyzed. (**B**) Antigen-specific IgG titer. (**C**) PsV neutralization titer (NAT_50_) against B.1.1.7 and B.1.351 pseudovirus (PsV). Bars show GMT ± SD. Data were log transformed and analyzed by Student’s *t* test (unpaired, two-tailed). (**D**) Inhibition rates of immune serum against the B.1.1.7 and B.1.351 PsV. Bars show mean ± SD. The results are representative of 2 independent experiments. Vac is an abbreviation for Vaccine. Dotted line defines the limit of detection.

The immunogenicity of SCTV01C and the two monovalent vaccines were further evaluated in C57BL/6J mice with a two-dose prime-boost regimen at a dosage of 1 μg /antigen, based on dose–response study of SCTV01C and its two corresponding monovalent vaccines (Figure 1). Seven days after the booster immunization, sera were analyzed for antigen-specific antibody titers and neutralizing activities (Figure 2A).

The SCTV01C bivalent vaccine induced a significantly higher specific IgG titer (Geometric Mean Titer, GMT) to the B.1.1.7 antigen (GMT = 1024000) than the B.1.1.7 monovalent vaccine (GMT = 430539) (*p* = 0.0013), and a slight increase in specific IgG titer to the B.1.351 antigen (GMT = 1448155) than the B.1.351 monovalent vaccine (GMT = 1116680) (*p* = 0.1202) (Figure 2B). Conversely, more significant increases in the cross-neutralizing antibody titers induced by SCTV01C against the variant not included in the monovalent vaccines were observed (Figure 2C,D). As shown in Figure 2C, the bivalent vaccine SCTV01C induced strong and balanced neutralizing antibody titers (NAT_50_) against both vaccine variants (B.1.1.7 NAT_50_ = 13019; B.1.351 NAT_50_ = 10480), while the B.1.1.7 monovalent vaccine only induced a strong neutralizing antibody titer against the B.1.1.7 vaccine variant (NAT_50_ = 9412) but its cross-neutralizing antibody titer against the non-vaccine variant B.1.351 was 20-fold lower (NAT_50_ = 489). Similarly, the B.1.351 monovalent vaccine showed a 7.6-fold lower cross-neutralizing antibody titer against the non-vaccine variant B.1.1.7 (NAT_50_ = 2131) than the vaccine variant B.1.351 (NAT_50_ = 16106). The above results demonstrated a clear advantage of the bivalent vaccine over the two monovalent vaccines in inducing a more balanced neutralizing antibody response to the two vaccine strains.

### 3.3. Cross-Neutralizing Activities Elicited by the B.1.1.7 Monovalent, B.1.351 Monovalent and SCTV01C Bivalent Vaccines

**Figure 3:**
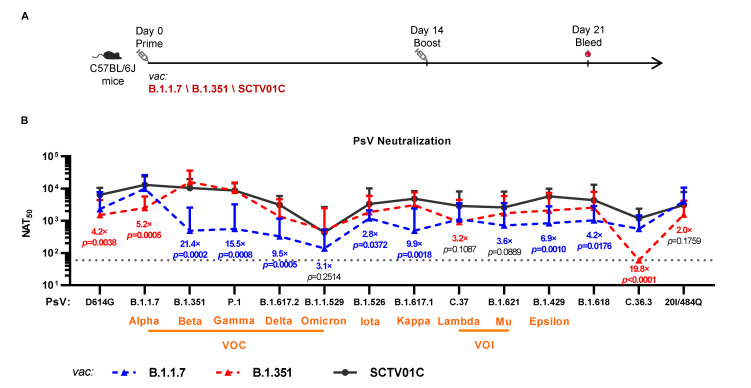
Cross-neutralizing activities of the B.1.1.7 monovalent, B.1.351 monovalent and SCTV01C bivalent vaccines against SARS-CoV-2 pseudovirus (PsV) based on D614G, VOCs, VOIs, VUMs and other variants. (**A**) Scheme of immunization and serum collection. C57BL/6J mice (n = 8/group) were immunized with B.1.1.7 monovalent vaccine (1 μg/dose), B.1.351 monovalent vaccine (1 μg/dose), or SCTV01C (1 μg/dose for each antigen) on days 0 and 14. Inhibition of PsV infection by serially diluted mouse sera were analyzed on day 21. (**B**) Neutralizing antibody titers against each variant lineage were analyzed. The results are representative of two independent experiments. Dotted line defines the limit of detection (represented as a GMT of 60). VOC, variants of concern; VOI, variants of interest. Vac is an abbreviation for Vaccine. Bars show GMT ± SD. Data were log transformed and analyzed by Student’s *t* test (unpaired, two-tailed).

In addition to the neutralizing antibody titers measured against the Alpha and Beta variants, cross-neutralizing antibody titers against 12 other variants, including the D614G variant, VOCs (P.1, B.1.617.2, B.1.1.529), VOIs (C.37, B.1.621), variants under monitoring (VUMs) (B.1.526, B.1.617.1, B.1.429, C.36.3), and two other variants (B.1.618, 20I/484Q) were also evaluated (Figure 3). The fold-reductions of the cross-neutralizing antibody titers against the non-vaccine variants in comparison to the vaccine variant for both B.1.1.7 and B.1.351 monovalent vaccines are listed in Table 1.

In the case of the B.1.1.7 monovalent vaccine, the neutralizing antibody titers against the B.1 (D614G) and 20I/484Q variants were 2323 and 4040, respectively, representing a reduction of 4.1-fold and 2.3-fold from the NAT_50_ against the B.1.1.7 variant. A 7.9-fold to 28.7-fold reduction was observed for the other 10 variants. The NAT_50_ against the B.1.1.529 (Omicron) was 139, the lowest among all 14 variants, representing a 67.8-fold reduction as compared to the NAT_50_ against the B.1.1.7 variant (Table 1).

In the case of the B.1.351 monovalent vaccine, a 10.7-fold reduction in neutralizing antibody titer against the D614G variant (NAT_50_ = 1511) was observed as compared to the B.1.1.7 vaccine’s four-fold reduction. Conversely, the B.1.351 monovalent vaccine exhibited only a slight reduction (1.9-fold) in the NAT_50_ against the P.1 variant (NAT_50_ = 8644), as compared to the B.1.1.7 vaccine’s 16.7-fold reduction. Similar results have been reported based on a trimeric B.1.351 S-ECD protein vaccine-immunized mouse sera [32]. The mild decrease in neutralizing activity of the B.1.351 vaccine-immunized sera against the P.1 variant might attribute to the similar mutation patterns in RBD between the B.1.351 (K417N, E484K, N501Y) and P.1 (K417T, E484K, N501Y) variants [33]. This finding is supported by a published report that the neutralizing antibody titers of B.1.351-infected human sera against the P.1 PsV were also comparable to that against the B.1.351 PsV [34]. Likewise, the B.1.351 monovalent vaccine elicited 5.2-fold to 18.1-fold reductions in NAT_50_ against the other VOCs, VOIs and VUMs, and no detectable neutralizing activity against the C.36.3 variant was observed (Table 1).

Among the 14 variants evaluated, the B.1.1.7 monovalent vaccine induced higher NAT_50_ against five variants, while the B.1.351 monovalent vaccine induced higher NAT_50_ against the other nine variants. Apparently, the B.1.351 variant seems to be a better monovalent vaccine candidate against viral variants. Nevertheless, neither monovalent vaccine was able to induce potent and balanced cross-neutralizing antibody titers against all 14 variants, and hence may not be an ideal second generation vaccine candidate. The limited cross-neutralizing activities of monovalent vaccines against diverse variants might be compensated by a multivalent vaccine, as different variants could offer broad coverage on the key mutations in the RBD region. This is supported by published results that broad immunity against wild type (WT) as well as B.1.1.7, P.1, and B.1.351 variants in a murine model was induced by an antigen representing multiple SARS-CoV-2 variants [35].

Conversely, the bivalent vaccine SCTV01C induced potent and broad cross-neutralizing antibodies against all 12 non-vaccine variants (Figure 3 and Table 1). In comparison to the two monovalent vaccines, SCTV01C showed equivalent levels of NAT_50_ against the P.1, B.1.1.529 and 20I/484Q variants but significantly higher NAT_50_ against the remaining nine variants. The NAT_50_ against the B.1.1.529 variant was 437, the lowest NAT_50_ among the titers against all 14 variants measured, due to the fact that the B.1.1.529 variant carries 25 distinctive mutations when compared with those mutations harbored in the S protein of other VOCs [36]. The above results clearly demonstrated significant advantages of SCTV01C over the two monovalent vaccines and its potential application as a second generation vaccine candidate with cross-neutralizing activities against existing variants and variants of potential concern emerging in the future.

**Table 1:** PsV neutralizing antibody titer elicited by B.1.1.7 monovalent, B.1.351 monovalent, and SCTV01C bivalent vaccines.

Vaccines	Variants	D614G	VOC					VOI		VUM					
		B.1	B.1.1.7	B.1.351	P.1	B.1.617.2	B.1.1.529	C.37	B.1.621	B.1.526	B.1.617.1	B.1.429	C.36.3	B.1.618	20I/484Q
		/	(Alpha)	(Beta)	(Gamma)	(Delta)	(Omicron)	(Lambda)	(Mu)	(Iota)	(Kappa)	(Epsilon)	/	/	/
**B.1.1.7 Vaccine**	**NAT**_50_	2323	9412	489	563	328	139	1114	720	1194	490	840	563	1027	4040
	**Fold Reduction ^a^**	4.1	1.0	19.2	16.7	28.7	67.8	8.4	13.1	7.9	19.2	11.2	16.7	9.2	2.3
**B.1.351 Vaccine**	**NAT**_50_	1511	2131	16,106	8644	1364	519	913	1726	1848	3071	2073	60 ^c^	2570	1574
	**Fold Reduction ^b^**	10.7	7.6	1.0	1.9	11.8	18.1	17.6	9.3	8.7	5.2	7.8	268.4	6.3	10.2
**SCTV01C Bivalent Vaccine**	**NAT**_50_	6411	13,019	10,480	8745	3134	437	2892	2595	3324	4846	5761	1185	4365	3134
	**Fold Increase** **(vs B.1.1.7 vac)**	2.8	1.4	21.4	15.5	9.6	3.1	2.6	3.6	2.8	9.9	6.9	2.1	4.2	0.8
	**Fold Increase** **(vs B.1.351 vac)**	4.2	6.1	0.7	1	2.3	0.8	3.2	1.5	1.8	1.6	2.8	19.8	1.7	2

^a^, compared with NAT_50_ against the B.1.1.7 PsV. ^b^, compared with NAT_50_ against the B.1.351 PsV. ^c^, lower than the limit of detection (LOD, represented as a GMT of 60). VOC, variants of concern; VOI, variants of interest; VUM, variants under monitoring.

### 3.4. T Cell Responses Induced by the B.1.1.7 Monovalent, B.1.351 Monovalent and SCTV01C Bivalent Vaccines

Vaccine-induced T cell immunity, especially a Th1-biased immune response, is crucial for the COVID-19 vaccine protective efficacy. A Th2-biased immune response is considered to be the major cause for vaccine-associated enhanced respiratory disease (VAERD), as shown by a preclinical study of an inactivated SARS-CoV vaccine, as well as clinical research of a respiratory syncytial virus (RSV)-inactivated vaccine [37,38].

**Figure 4:**
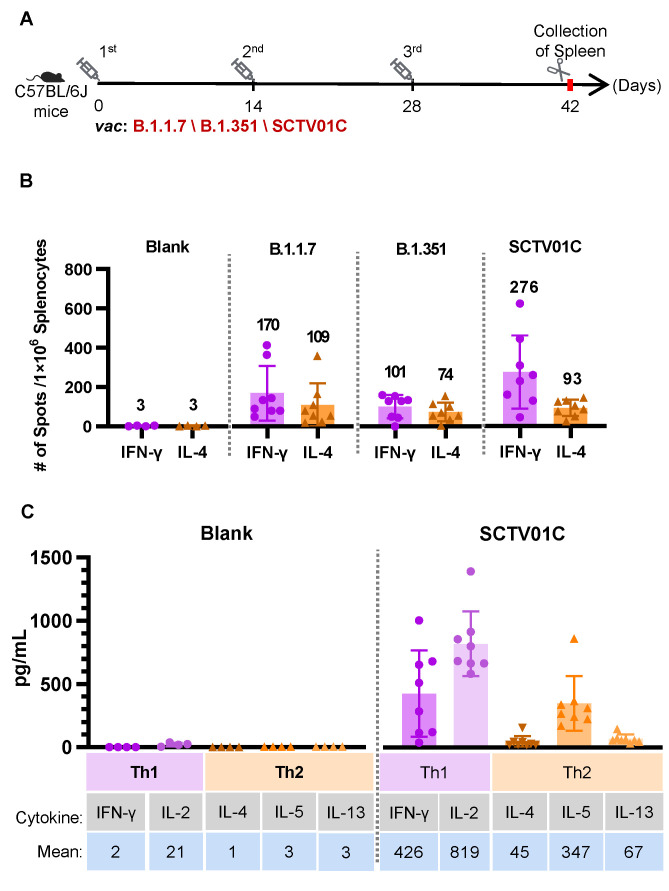
Th1 responses induced by the B.1.1.7 monovalent, B.1.351 monovalent and SCTV01C bivalent vaccines. (**A**) Scheme of immunization and tissue processing. C57BL/6J mice (n = 8/group) were immunized with B.1.1.7 monovalent vaccine (1 μg/dose), B.1.351 monovalent vaccine (1 μg/dose), or SCTV01C (1 μg/dose for each antigen) on days 0, 14 and 28. Splenocytes from immunized mice were stimulated with SARS-CoV-2 spike protein S polypeptides on day 42. (**B**) Number of IFN-γ (Th1 cytokine) and IL-4 (Th2 cytokine) positive Spot-forming cells (SFC) was evaluated. (**C**) Th1 (IFN-γ, IL-2) and Th1 (IL-4, IL-5 and IL-13) cytokines in culture supernatants from S peptides-stimulated mouse splenocytes were measured by flow cytometry. The results are representative of 3 independent experiments. Vac is an abbreviation for Vaccine. Bars show mean ± SD.

To evaluate the cellular immune response, splenocytes from vaccinated mice were used for detection of IFNγ-positive (representing Th1 immune response) or IL-4-positive (representing Th2 immune response) splenocytes after stimulating with SARS-CoV-2 spike protein S polypeptides (2 μg/mL) using ELISpot assays. SCTV01C and the two monovalent vaccines induced significant T cell responses as indicated by the number of spot-forming cells (SFC) (Figure 4B). The ratios of IFNγ^+^ SFCs/IL-4^+^ SFCs in each group were 2.38, 1.31, 3.96 (Figure 2C), suggesting the T cell responses induced by the three vaccines were all Th1-biased. Th1-biased responses induced by SCTV01C were further validated by concentrations of Th1 (IFNγ, IL-2) and Th1 (IL-4, IL-5 and IL-13) cytokines in culture supernatants from SCTV01C vaccinated mouse splenocytes in the presence of S peptides (Figure 4C).

### 3.5. Humoral Immune Responses Induced by Booster Dose of SCTV01C

**Figure 5:**
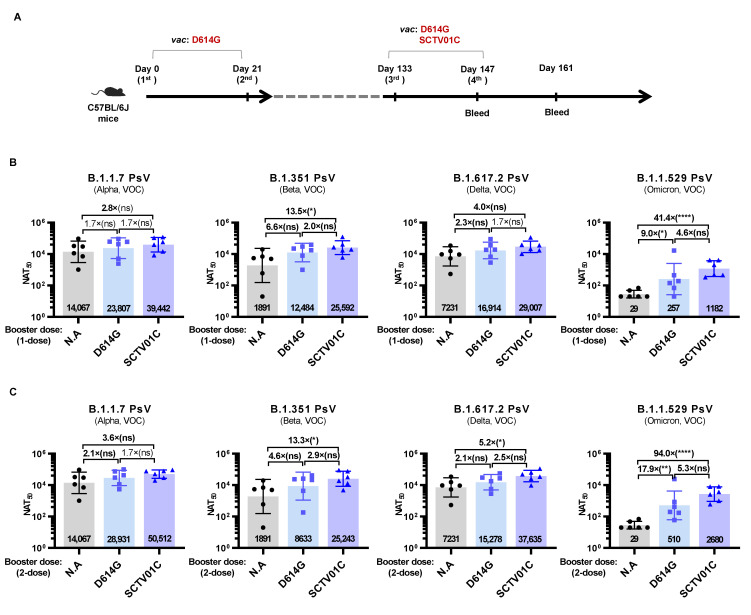
Humoral immune responses induced by SCTV01C or the D614G vaccine as booster shots in mice previously immunized with a 2-dose scheme of D614G vaccine. (**A**) Scheme of immunization and serum collection. C57BL/6J mice (n = 6/group) were immunized with a 2-dose scheme of D614G vaccine (0.5 μg/dose) on days 0 and 21. On days 133 and 147, mice received two booster shots of SCTV01C (Mix Regimen; 0.5 μg/dose/antigen) or the D614G vaccine (D614G Regimen; 0.5 μg/dose). Neutralizing antibody titers against B.1.1.7, B.1.351, B.1.617.2 and B.1.1.529 PsV were analyzed on day 147 (**B**) and day 161 (**C**). The results are representative of 2 independent experiments. Bars show GMT ± SD. Data were log transformed and analyzed by Student’s *t* test (unpaired, two-tailed). * *p* < 0.05, ** *p* < 0.01, **** *p* < 0.0001. ns: not statistically significant.

The necessity of the COVID-19 vaccine booster shots is widely recognized due to waning vaccine immunity over time. A D614G variant-based monovalent vaccine was used to mimic the original strain-based first-generation vaccines. C57BL/6J mice previously immunized with a two-dose schedule of the D614G vaccine received two booster shots of SCTV01C (Mix Regimen) or the D614G vaccine (D614G Regimen), and neutralizing antibody titers against the PsV of B.1.1.7, B.1.351, B.1.617.2 and B.1.1.529 variants were analyzed 14 days after the third or the fourth immunization (Figure 5A).

Two weeks after the second immunization with the D614G vaccine (day 35), the NAT_50_ against the B.1.1.7 PSV reached 14067, with a slight decrease in NAT_50_ against the B.1.617.2 PSV (NAT_50_ = 7231, 1.9-fold reduction) and a sharp decrease in NAT_50_ against the B.1.351 (NAT_50_ = 1891, 7.5-fold reduction), while no detectable neutralizing activities were observed against the B.1.1.529 PSV (Figure 5B). Two weeks after the first booster shot (day 147), elevated neutralizing activities were observed in both regimens against all four variants tested. The Mix Regimen induced higher NAT_50_ against the B.1.1.7 (1.7-fold), B.1.351 (2.0-fold), B.1.617.2 (1.7-fold) and B.1.1.529 (4.6-fold) variants than the D614G Regimen (Figure 5B). Two weeks after the second booster shot (day 161), neutralizing activities of both regimens against B.1.1.7, B.1.351 and B.1.617.2 showed no significant increase, while a further increase in NAT_50_ against the B.1.1.529 PSV was observed in both the D614G Regimen and Mix Regimen (two-fold increase) (Figure 5C). The Mix Regimen exhibited the highest levels of NAT_50_ against all four variants versus the D614G Regimen, suggesting the potential application of SCTV01C as a booster shot for the population previously immunized with the original strain-based first-generation vaccines or previously infected by original strain of SARS-CoV-2.

## 4. Discussion

More than 15 first-generation vaccines developed based on the original strain of SARS-CoV-2 have been approved or authorized for emergency use, including two mRNA vaccines, two subunit vaccines, four viral vector vaccines and seven inactivated vaccines [39]. However, the emergence of a large number of SARS-CoV-2 variants with mutations in key neutralizing epitopes not only increases the transmissibility but also reduces the protective effect of the first-generation vaccines and the effectiveness of the therapeutic antibodies based on the original strain of SARS-CoV-2 [33,40,41,42,43,44,45]. Thus, variant-tailored second-generation vaccines, especially those with broadly protective efficacy, might be an effective approach to defend against the pandemic.

Among the VOI and VOC variants designated prior to the emergence of the new variant Omicron (B.1.1.529), the B.1.351 variant showed the most significant impact on the efficacy of first-generation vaccines [40,43,46], and has been the main focus for the development of second-generation vaccines against SARS-CoV-2 variants [32,47]. In this study, the B.1.351 monovalent vaccine induced potent neutralizing activity against the B.1.351 variant, with an equivalent NAT_50_ against the P.1 variant, which may be attributed to the fact that both B.1.351 and P.1 contain the K417N/T, E484K, and N501Y mutations [33]. This is consistent with previous studies based on the trimeric S-ECD vaccine [32] and B.1.351-infected sera [34].

However, the neutralizing activity of the B.1.351 monovalent vaccine against the other 13 major variants were markedly decreased, in particular, and no neutralizing activity against the C.36.3 variant was detected. The B.1.1.7 monovalent vaccine also showed severely reduced neutralizing activities against most of the variants tested. These data indicate that vaccines based on a single variant may not be sufficient to induce cross-neutralizing activities. Ancestral strain-based vaccines reported to exhibit relatively broad neutralizing activities against SARS-CoV-2 variants also showed relatively diminished cross-reactivity against variants [48,49,50].

A preliminary study has indicated that antigens representing multiple virus strains can generate broad-spectrum immunity against WT as well as VOCs (such as B.1.1.7, P.1, and B.1.351) in mouse models [35], and immunization with multiplexed chimeric spikes can potentially induce broader protection against Sarbecoviruses [51]. Multivalent vaccines, with broad coverage on the key mutations in the spike protein of various SARS-CoV-2 variants, might induce more balanced cross-neutralizing activities. Protein-based or RNA-based bivalent vaccines, composed of antigens originating from the ancestral strain and variant strain of SARS-CoV-2, showed balanced neutralizing activities against both antigen-matched strains in mouse models [32,47,52,53]. Some of the candidates of the bivalent vaccines have entered the clinical stage for further assessment of efficacy against COVID-19 variants (NCT04927065, NCT05004181, NCT04889209, NCT05029856, NCT04904549); however, cross-neutralization activities of these vaccines against antigen-mismatched variants have not been elaborated in preclinical studies thus far.

In this study, we evaluated the neutralizing activity of variants Alpha- and Beta-based bivalent vaccine SCTV01C against 14 SARS-CoV-2 variants, including VOCs, VOIs and VUMs. SCTV01C induces potent and relatively balanced broad-spectrum humoral immune responses against all 14 variants tested, with potential advantages over the two corresponding monovalent vaccine (Figure 3 and Table 1). Furthermore, strong Th1-biased T cell responses were also observed in the mouse immunogenicity studies (Figure 4). Our data also demonstrated potential application of SCTV01C as a booster shot post immunization with the first-generation vaccines (Figure 5).

The protein-based COVID-19 vaccines are well tolerated, and thus far, no major side effects have been reported [54,55,56]. Squalene-based oil-in-water adjuvants, such as AS03 and MF59, have been approved and widely used in influenza vaccines with favorable safety profiles [57,58,59,60]. Preclinical toxicity studies of SCTV01C did not show any unexpected safety concerns [30].

The current study has potential limitations. First, due to the requirement for a BSL-3 facility, SCTV01C-vaccinated mouse sera were not used for assessment of neutralization activities against live viruses; however, this will be evaluated in clinical stages with SCTV01C-vaccinated human sera. Second, the protection efficacy of SCTV01C against antigen-matched or antigen-mismatched variants needs to be further evaluated in mouse challenge model.

In summary, our data suggest that the bivalent vaccine SCTV01C could induce broad cross-neutralizing immune responses against SARS-CoV-2 VOCs, VOIs, and VUMs, as well as potent Th1-biased T cell responses. Clinical studies of SCTV01C in previously unvaccinated and vaccinated populations are needed to further evaluate its application in controlling the COVID-19 pandemic.
